# Effects of short-term very low-calorie diet on metabolic profile in patients with metabolic syndrome

**DOI:** 10.3389/fendo.2025.1671870

**Published:** 2025-12-11

**Authors:** Yi Zhou, Shu-nan Li, Tianchi Hu, Shang-qing Hu, Na-fen Li, Yan-jing Fan, Jing-wen Yu, Yuan Yuan, Ying-xin Chen, Min-xing Sun, Hong-hua Chen, Bo Li, Qi-da He

**Affiliations:** 1 Department of Endocrinology, Xiamen Hospital of Traditional Chinese Medicine, Xiamen, China; 2 Department of General Medicine, School of Health, Fujian Medical University, Fuzhou, China; 3 Department of Traditional Chinese Medicine, The First Affiliated Hospital of Xiamen University, School of Medicine, Xiamen University, Xiamen, China; 4 Xiamen Clinical Nutrition Quality Control Center, Xiamen, China; 5 Fujian University of Traditional Chinese Medicine, Fuzhou, China

**Keywords:** very low-calorie diet, metabolic syndrome, 1H-NMR, metabolism, metabolic profile

## Abstract

**Background:**

Accumulating evidence indicates that dietary modifications confer beneficial effects on metabolic syndrome (MetS). In clinical practice, short-term very low-calorie diets (VLCD) have been established as an effective intervention for improving MetS, even in the absence of exercise. However, the impact of a short-term VLCD on the serum metabolic profile of patients with MetS remains to be elucidated.

**Methods:**

18 adult patients meeting the diagnostic criteria for MetS were enrolled and placed on a 9-day VLCD regimen. Anthropometric parameters, blood pressure, and lipid metabolism indices were measured before and after the intervention. Serum metabolic profiles were subsequently acquired using ^1^H-NMR spectroscopy.

**Results:**

Following the short-term VLCD intervention, patients with MetS exhibited significant reductions in body weight, waist circumference, and BMI (*P* < 0.05). Blood pressure was also significantly lowered (*P* < 0.05). Furthermore, the intervention regulated both glucose and lipid metabolism. Serum metabolomic analysis identified 20 characterized metabolites, all of which showed significantly decreased levels post-treatment (*P* < 0.05). Pathway analysis indicated that the short-term VLCD modulated key metabolic pathways involved in energy and lipid metabolism, insulin sensitivity, anti-inflammatory and antioxidant responses, cellular signaling, and neurohormonal regulation.

**Conclusions:**

Our study demonstrates that a short-term VLCD is an effective and safe intervention for improving anthropometric parameters, blood pressure, and lipid metabolism in patients with MetS. The observed therapeutic effects appear to be mediated through a remodeling of the serum metabolic profile and a concomitant modulation of key metabolic pathways. These findings provide a scientific rationale for the clinical application of short-term VLCD in MetS management.

## Introduction

Metabolic syndrome (MetS) is characterized by the convergence of various metabolic risk factors. The diagnosis of MetS requires the concurrent presence of at least three of the following components: central adiposity, dyslipidemia, dysglycemia, and elevated blood pressure (1). Despite its well-defined clinical presentation, MetS remains a considerable management challenge. Its global prevalence, which has reached 20-25% in many developed nations (9, 10), is rising and poses a significant public health burden (2, 3). This is of particular concern as MetS substantially elevates the risk of type 2 diabetes, cardiovascular disease, and other chronic conditions (4–8). In this context, dietary intervention has emerged as a cornerstone in the management of MetS (11).

As a therapeutic dietary intervention, a very low-calorie diet (VLCD) has garnered considerable attention in recent years (12). A VLCD denotes a nutrition plan with a caloric intake of 400–800 kcal daily, wherein carbohydrates contribute approximately 50% of caloric content, and proteins are provided at a dosage of 0.8-1.5 grams per kilogram per day (13). And the method needs to be supplemented with essential fatty acids, vitamins, and minerals. However, long-term VLCD is believed to potentially lead to malnutrition (14). Numerous investigations have demonstrated the efficacy of short-term VLCD in enhancing insulin sensitivity and addressing various constituents of the metabolic syndrome (12, 15, 16). Nevertheless, the underlying mechanisms by which short-term VLCD exerts its therapeutic effects on MetS remain to be fully elucidated.

Metabolomics technology was a powerful tool for studying the characteristic metabolites associated with diseases. Moreover, metabolomics technology has been widely applied in studies associated with metabolic syndrome. Hydrogen-1 nuclear magnetic resonance (^1^H-NMR), as one of the components of metabolomics technology, has the advantages of convenient detection and high sensitivity. Hence, ^1^H-NMR spectroscopy was applied to probe the impact of short-term VLCD on the metabolite profile associated with MetS in this study.

## Materials and methods

### Participants

This study is a single-arm pilot study without a control group, where the baseline clinical characteristics collected from all participants were compared before and after treatment. 18 patients diagnosed with MetS were enrolled from the Endocrinology Department of Xiamen Traditional Chinese Medicine Hospital from June 2023 to June 2024. All participants were vetted to ensure they satisfied the predefined inclusion and exclusion criteria. This study was meticulously conducted in adherence to the ethical principles enshrined in the Helsinki Declaration, and the ethical approval was reviewed and approved by the Medical Ethics Committee of Xiamen Traditional Chinese Medicine Hospital (Protocol Number: 2023-K029-01). The studies were conducted in accordance with the local legislation and institutional requirements. Written informed consent was obtained from all individual participants.

### Inclusion criteria

The following criteria were established for the inclusion of participants in the study: (i) all participants between the age of 18 to 65 years; (ii) central obesity (waist circumference of ≥90 cm in males or ≥80 cm in females, and at least two of the following factors: elevated triglyceride (TG) ≥1.7 mmol/L, high-density lipoprotein (HDL) <1.03 mmol/L for males and <1.29 mmol/L for females, systolic blood pressure (SBP) ≥130 mmHg or diastolic blood pressure (DBP) of ≥85 mmHg, fasting blood glucose (FBG) ≥5.6 mmol/L; (iii) the duration of both diabetes and hypertension should not exceed 5 years; (iv) all participants were required to provide informed consent and to confirm their voluntary participation in the study (17).

### Exclusion criteria

The study applied the following exclusion criteria to ensure the selection of appropriate participants: (i) females who were pregnant or lactating; (ii) acute and chronic diabetes complications; (iii) participants with severe liver or kidney dysfunction, significant cardiovascular diseases, chronic infections, thyroid dysfunction, malignant tumor or other serious health issues; (iv) individuals with neurogenic anorexia or malnutrition; (v) neurological and Psychiatric Conditions.

### Intervention methods

All participants were instructed to discontinue the use of hypoglycemic, lipid-lowering, and antihypertensive medications for one week. The subjects underwent a 9-day VLCD regimen. This regimen consisted of three phases: a 2-day buffer period at 600 kcal/day, a 5-day intensive phase (days 3-7) at 300 kcal/day, and a 2-day recovery phase (days 8-9) at 600 kcal/day. The diet maintained a consistent macronutrient distribution of 55% carbohydrates, 20% protein, and 25% fat throughout all phases (18). During the intervention, participants were advised to restrict physical activity to basic daily tasks and light exercise.

### Baseline clinical characteristics

Among the 18 participants included in this study, there were 10 males (55.6%) and 8 females (44.4%). Among the 18 participants, due to personal willingness and case dropout, serum metabolomics profiling was ultimately conducted on 14 participants, comprising 7 males (50%) and 7 females (50%). Before and after the short-term VLCD intervention, a comprehensive array of baseline clinical characteristics was assessed in the patients. These baseline clinical characteristics included anthropometric measurements such as body weight (BW), waist circumference (WC), BMI, body fat mass, body fat percentage, and blood pressure.

Fasting peripheral venous blood samples were collected after an 8–10 hour fast. Levels of fasting blood glucose (FBG) and fasting insulin (FINS) were quantified using a fully automated biochemical analyzer and a chemiluminescence immunoassay analyzer, respectively. The homeostasis model assessment of insulin resistance (HOMA-IR) was then calculated from FBG and FINS to evaluate insulin resistance. Lipid profiles, including total cholesterol (TC), triglycerides (TG), high-density lipoprotein (HDL), and low-density lipoprotein (LDL), were also analyzed. Additionally, the safety index of liver and kidney function was assessed.

### ^1^H-NMR metabolomics assays

Following an 8–10 hour fasting period, 3mL of peripheral venous blood was collected from each participant. The samples were centrifuged at 3000×g for 10min to isolate serum. A 400μL serum was then mixed with 200μL phosphate buffer solution and subjected to high-speed centrifugation at 13,000×g for 20min at 4°C. Subsequently, 500μL of the resulting supernatant was transferred to a 5mm NMR tube for analysis. Finally, all processed samples were detected by a ^1^H-NMR spectrometer (Bruker AVANCE-III 600MHz, Germany).

### Data processing and analysis

The ^1^H-NMR spectra of all samples underwent rigorous phase and baseline corrections using MestReNova v9.0.1 software. Subsequently, the data were imported into SIMCA-P 14.0 software. Orthogonal Partial Least Squares Discriminant Analysis (OPLS-DA) was then conducted to differentiate between the metabolic profiles of the samples. Finally, differential metabolites with VIP>1 and P<0.05 were pointed out for before and after treatment.

### Statistical analysis

SPSS software (version 26, IBM, United States) was applied for statistical analyses of clinical variables. The data were performed using paired t-tests for normally distributed parameters, while the Wilcoxon signed-rank test was employed for variables that deviated from normality. *P* < 0.05 was considered statistically significant. All statistical analyses were conducted using SPSS software(IBM, United States).

## Results

### Baseline clinical characteristics

Before and after the treatment of a short-term VLCD, the baseline clinical characteristics were observed. The results showed that levels of BW, WC, BMI, SBP, DBP, FBG, FINS, HOMA-IR, HDL, and TG were decreased significantly after treatment (*P* < 0.05). However, there was no significant alteration in the levels of body fat mass, body fat percentage, LDL and TC after treatment by short-term VLCD (*P*>0.05). Moreover, albumin (ALB), alanine aminotransferase (ALT), and creatinine (CR), which were related to the function of the liver and kidney, were not altered (*P*>0.05) (**Table 1**). Notably, AST was significantly increased after treatment by short-term VLCD (*P* < 0.05), but it remained in the normal range.

**Table 1:** Baseline clinical characteristics of all participants.

Parameters	Before treatment	After treatment	P value
Anthropometric parameters			
BW (kg)	90.43 ± 17.52	85.56 ± 16.64*	0.000
BMI (kg/m2)	32.28 ± 5.33	30.28 ± 5.02*	0.000
Body fat mass (kg)	34.78 ± 11.32	33.95 ± 10.91	0.307
Body fat percentage (%)	38.49 ± 6.15	37.85 ± 6.88	0.422
WC (cm)	101.89 ± 12.83	95.40 ± 12.42*	0.000
Blood pressure			
DBP (mmHg)	84.44 ± 11.64	74.44 ± 5.68*	0.003
SBP (mmHg)	134.11 ± 14.06	116.44 ± 8.10*	0.000
Glucose metabolism index			
FBG (mmol/L)	6.04 ± 2.27	4.29 ± 0.65*	0.001
FINS (μIU/mL)	25.29 ± 20.69	16.63 ± 16.20	0.043
HOMA-IR	6.52 ± 5.19	3.37 ± 3.66*	0.008
Lipid metabolism index			
HDL (mmol/L)	1.18 ± 0.22	1.03 ± 0.14*	0.003
LDL (mmol/L)	3.02 ± 0.77	2.99 ± 0.76	0.793
TC (mmol/L)	4.79 ± 0.99	4.52 ± 0.93	0.159
TG (mmol/L)	1.63 ± 0.71	0.99 ± 0.28*	0.001
Safety index			
ALB(g/L)	47.39 ± 9.14	44.33 ± 2.33	0.162
ALT (IU/L)	37.33 ± 20.53	37.11 ± 19.26	0.939
AST (IU/L)	24.78 ± 9.90	29 ± 9.75*	0.02
CR (μmol/L)	67.59 ± 13.37	67.61 ± 15.71	0.991

Data are presented as mean ± standard error. *Means the significantly different between before and after treatment. ALT, alanine aminotransferase; AST, aspartate aminotransferase; BMI, body mass index; BW, bodyweight; Cr, creatinine; Alb, albumin; DBP, diastolic blood pressure; FBG, fasting blood glucose; FINS, fasting insulin; HDL, high-density lipoprotein; HOMA-IR, homeostasis model assessment of insulin resistance; LDL, low-density lipoprotein; SBP, systolic blood pressure; TC, total cholesterol; TG, triglyceride; WC, waist circumference.

### Effects of short-term VLCD on serum metabolic profile in patients with MetS

**Figure 1:**
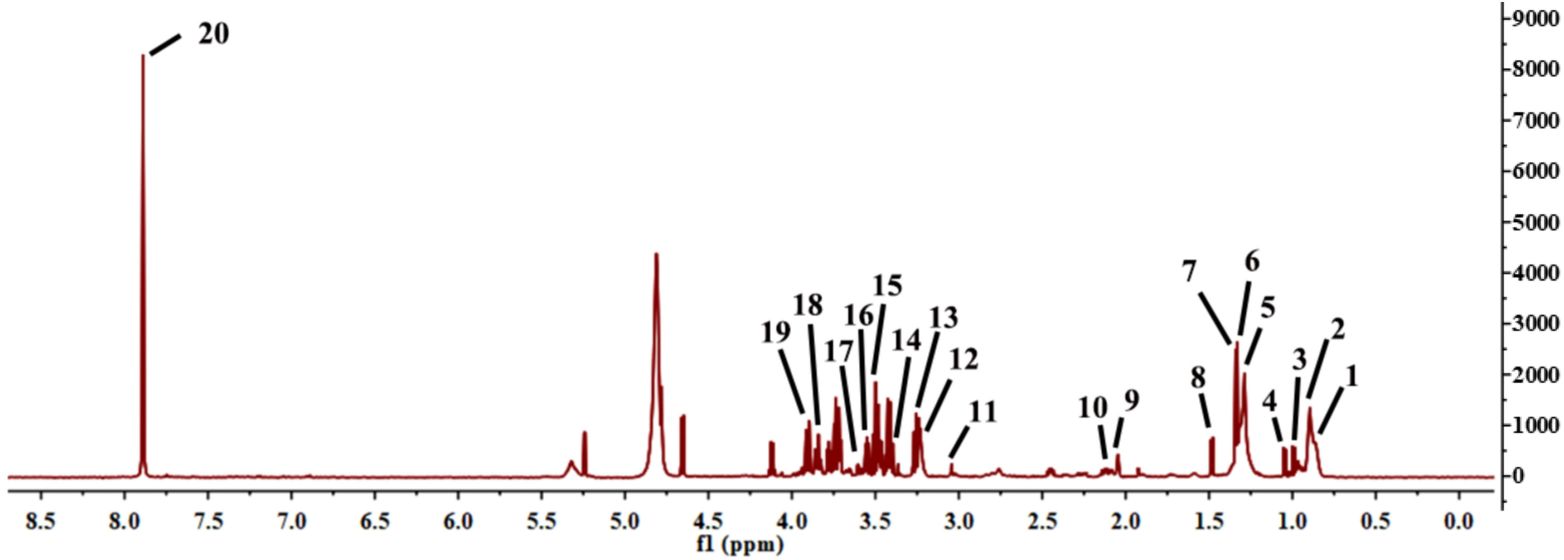
^1^H-NMR spectra of differential metabolites in serum. 1.Low density lipoprotein; 2.Very low-density lipoprotein; 3.Leucine; 4.Valine; 5.Diethyl methylmalonate; 6.Threonine; 7.Lactic acid; 8.Alanine; 9.Glutamic acid; 10.Acetone; 11.Phosphocholine; 12.Glycerol phosphocholine; 13.β-Glucose; 14.Betaine; 15.α-Glucose; 16.Glycine; 17.Glycerol; 18.Glutamine; 19.Serine; 20.Methylhistidine.

Although a total of 18 participants were enrolled in this study, there were only 14 participants who agreed to be assayed for serum metabolites. ^1^H-NMR was utilized to identify the characteristic metabolites in the serum. Meanwhile, the differential metabolites were fingered in the ^1^H-NMR spectra (**Figure 1**).

**Figure 2:**
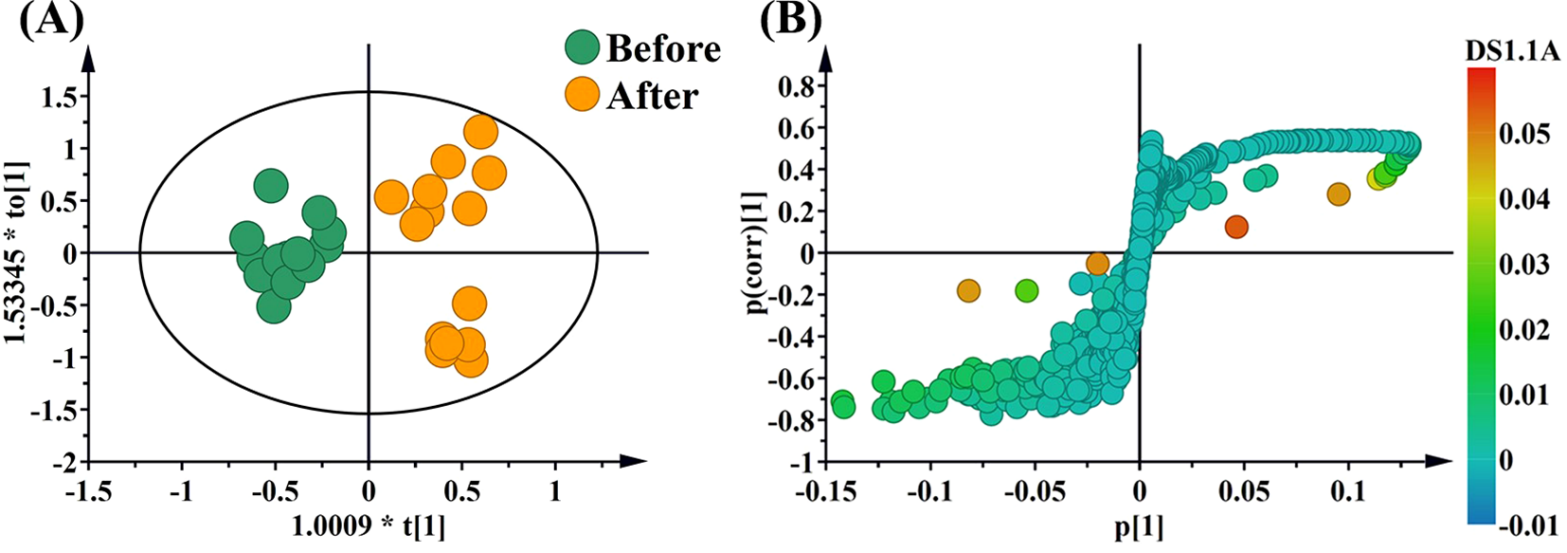
OPLS-DA and S-plots from the before treatment and after treatment. **(A)** means OPLS-DA; **(B)** means S-plots.

To point out biomarkers that reflect changes in metabolite profiles before and after the short-term VLCD, an OPLS-DA model was employed for a more in-depth analysis. Obviously, there was a significant discrete between the before and after treatment by short-term VLCD (**Figure 2**). The results shown that the samples from the two groups did not exhibit any crossover or significant overlap, indicating a clear separation and good dispersion (*P* < 0.05). This finding was crucial as it points to specific metabolites that may serve as biomarkers for the metabolic changes induced by the short-term VLCD treatment.

**Figure 3:**
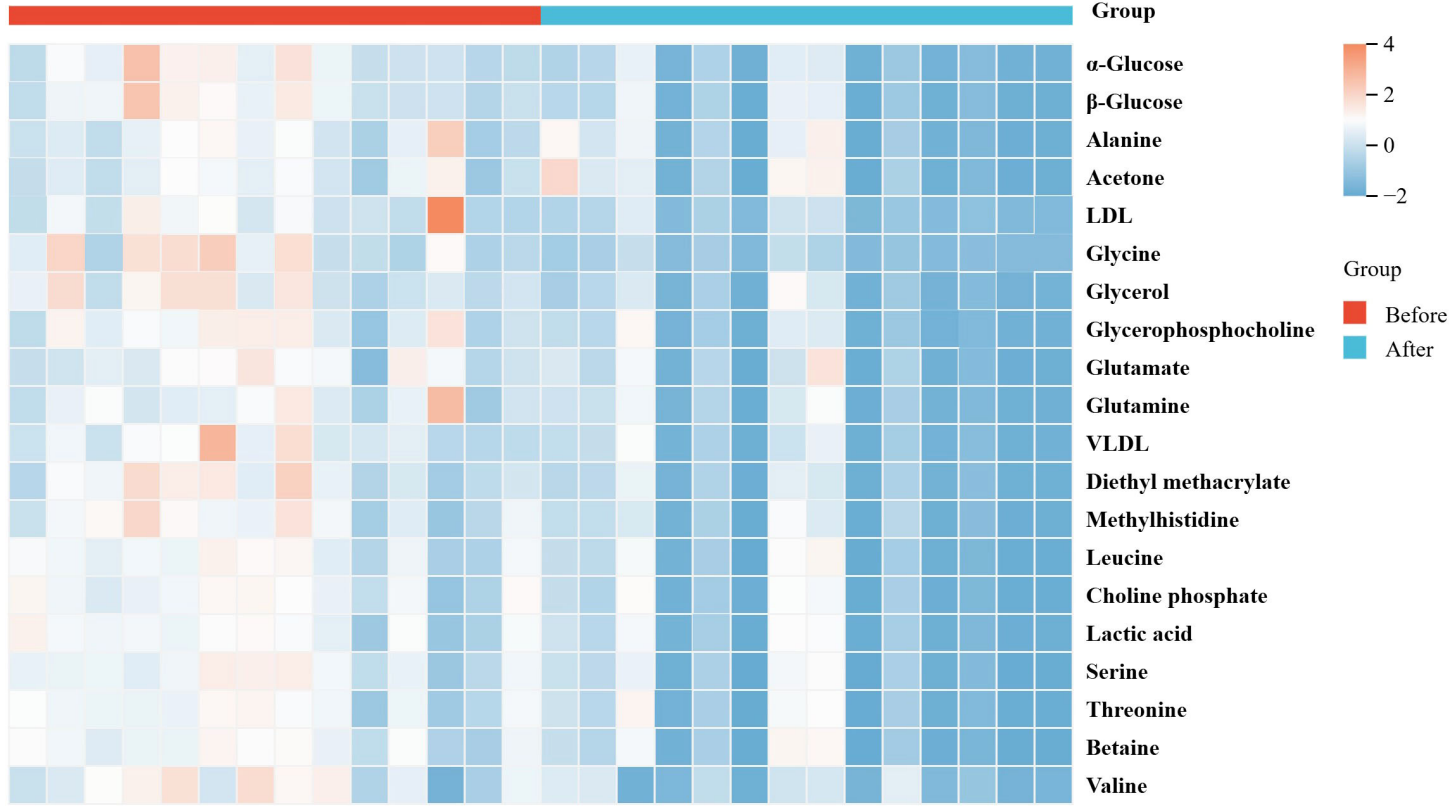
The heat-map of differential metabolites.

**Figure 4:**
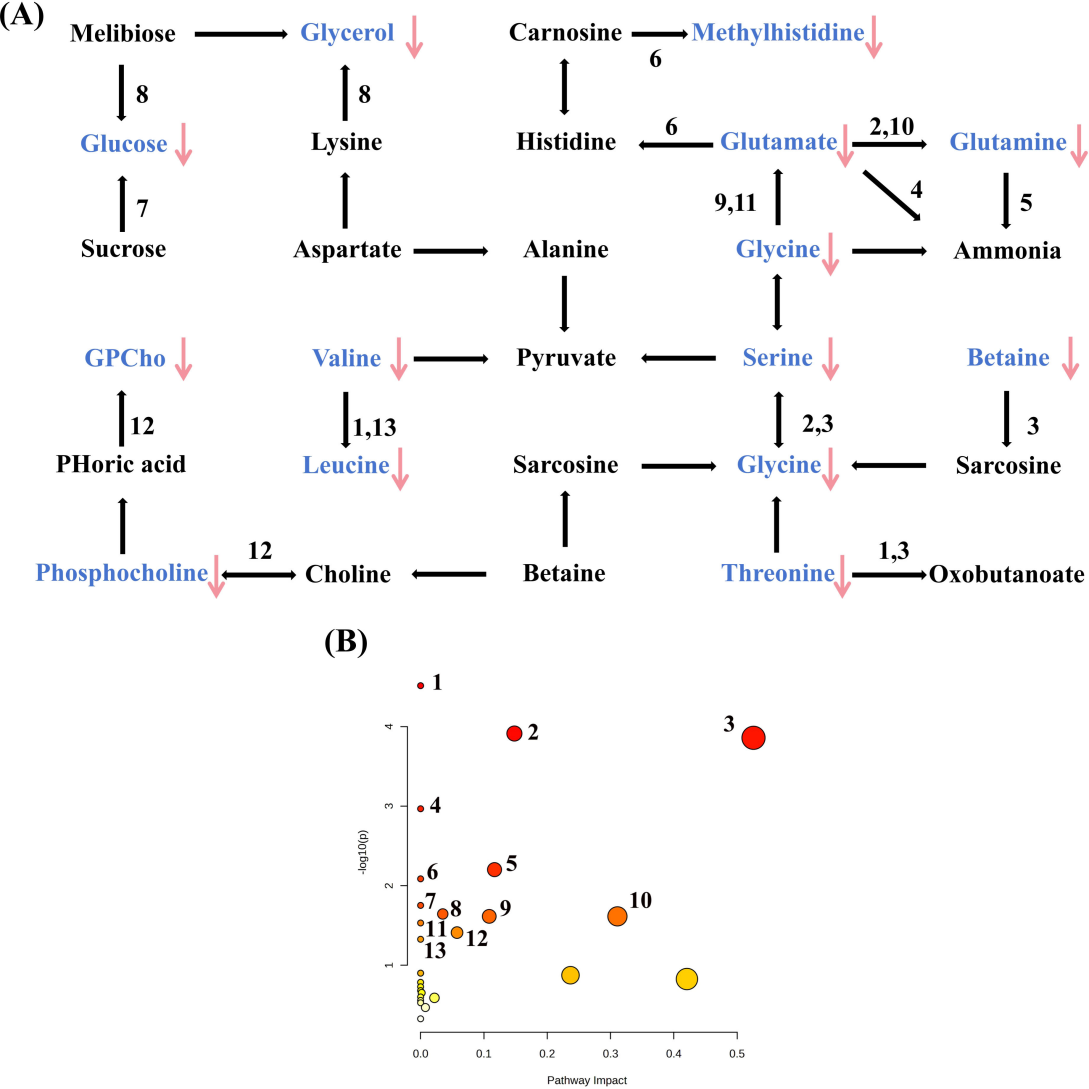
The metabolic pathways of differential metabolites. Blue means the differential metabolites. **(A)** means the metabolic pathway; Red arrow means reduced levels after treatment. Arrows mean a decrease in the level of metabolites. **(B)** means the scatter plot of the metabolic pathway;1.Valine, leucine and isoleucine biosynthesis; 2.Glyoxylate and dicarboxylate metabolism; 3.Glycine, serine and threonine metabolism; 4.Nitrogen metabolism; 5.Arginine biosynthesis; 6.Histidine metabolism; 7.Neomycin, kanamycin and gentamicinbiosynthesis; 8.Galactose metabolism; 9.Glutathione metabolism; 10.Alanine, aspartate and glutamate metabolism; 11.Porphyrin metabolism; 12.Glycerophospholipid metabolism; 13.Valine, leucine and isoleucine degradation.

Serum metabolomic profiling identified 20 differential metabolites that were significantly altered following the short-term VLCD intervention in patients with MetS (P<0.05). All identified differential metabolites were including acetone, alanine, betaine, diethyl methylmalonate, α-glucose, β-glucose, glycerophosphocholine, glutamate, glutamine, glycerol, glycine, lactic acid, low-density lipoprotein, methyl histidine, phosphatidylcholine, very low-density lipoprotein, leucine, serine, threonine, and valine, were significantly decreased after treatment (**Table 2**, **Figure 3**). Subsequently, KEGG enrichment analysis of these metabolites revealed 13 major metabolic pathways that were significantly enriched, based on a threshold of -log(p)>1 (**Figure 4**).

**Table 2:** Differential metabolic markers in serum.

No.	Metabolite	Chemical shift	VIP index	Log2 fold change	Trend
1	α-Glucose	3.54	1.38	0.34	Down
2	β-Glucose	3.25	1.39	0.40	Down
3	Alanine	1.48	1.34	0.40	Down
4	Acetone	2.23	1.26	0.46	Down
5	Betaine	3.27	1.45	0.34	Down
6	Diethyl methylmalonate	1.23	1.74	0.39	Down
7	Glutamate	2.13	1.05	0.39	Down
8	Glycerophosphocholine	3.23	1.98	0.40	Down
9	Glycine	3.56	1.51	0.31	Down
10	Glycerol	3.57	1.61	0.35	Down
11	Glutamine	3.78	1.36	0.35	Down
12	LDL	0.86	1.72	0.43	Down
13	Leucine	0.96	1.06	0.28	Down
14	Lactic acid	1.33	2.67	0.43	Down
15	Methyl histidine	7.78	1.05	0.54	Down
16	Phosphatidylcholine	3.22	1.41	0.38	Down
17	Serine	3.83	1.26	0.34	Down
18	Threonine	1.33	2.67	0.43	Down
19	VLDL	0.89	2.47	0.37	Down
20	Valine	0.99	1.06	0.22	Down

## Discussion

This study revealed that a short-term VLCD could significantly improve various parameters in patients with MetS, including BW, WC, BMI, SBP, DBP, FBG, FINS, HOMA-IR, HDL and TG. The findings suggest that a short-term VLCD treatment could effectively alleviate conditions such as hyperglycemia, hypertension, hypertriglyceridemia, overweight, and insulin resistance in MetS patients.

Notably, short-term VLCD treatment resulted in a reduction of HDL levels. The underlying mechanism might involve a sudden decrease in caloric intake, which triggers a stress response and subsequently leads to the suppression of HDL synthesis. Additionally, inadequate fat intake could also play a role by impeding HDL production (19, 20). Consequently, the observed short-term decline in HDL levels may signify a dynamic adaptive response of the body to acute energy deprivation, rather than a deterioration of its cardiovascular protective capabilities. Nevertheless, this hypothesis necessitates further validation. Additionally, the results demonstrated that a short-term VLCD treatment had no adverse effects on liver and kidney function. However, the elevated levels of AST imply that prolonged adherence to a VLCD might potentially impair liver function. Collectively, these findings support the notion that a short-term VLCD treatment is a more suitable therapeutic approach for MetS patients compared to a long-term VLCD.

MetS is intricately linked to multiple organs and metabolic targets, aligning seamlessly with the principles of metabolomics. The utilization of ^1^H-NMR spectroscopy enables the detection of numerous metabolites without the need for intricate sample preparation protocols, yielding high-resolution data. Following short-term VLCD treatment, the serum metabolic profile of MetS patients exhibited marked alterations. Notably, these differentially expressed metabolites are closely associated with key metabolic pathways, including energy-related metabolism, lipid-related metabolism, insulin sensitivity-related metabolism, inflammation-related metabolism, oxidative stress-related metabolism, cell signaling, and neurotransmitter-related metabolism. Below, we provide a detailed analysis of each differential metabolite identified:

### Energy-related metabolism

A short-term VLCD treatment is recognized as an effective approach for weight reduction through caloric restriction, potentially enhancing energy metabolism. Implementation of a short-term VLCD treatment results in decreased blood glucose levels, thereby reducing the dependency on glucose as a primary energy source. Moreover, this dietary intervention may enhance insulin sensitivity and diminish insulin resistance, facilitating more efficient insulin utilization and promoting glucose uptake into cells. Lactic acid can be integrated into the tricarboxylic acid (TCA) cycle either via gluconeogenesis or through the enzymatic conversion of lactate to pyruvate by lactate dehydrogenase and pyruvate dehydrogenase, thereby contributing to ATP synthesis (21). Similarly, acetone and glycerol can be metabolized to generate energy (22, 23). Leucine and valine not only serve as energy substrates but also enter the TCA cycle, providing additional energy for physiological processes (24, 25). Threonine participates in protein synthesis and gluconeogenesis, aiding in the maintenance of energy equilibrium (26). Metabolic disturbances are widely acknowledged as a hallmark feature of patients with MetS (17). Notably, short-term VLCD treatment can modulate key aspects of energy metabolism, including glucose, lactate, acetone, and glycerol pathways. The findings of this study demonstrate that short-term VLCD treatment facilitates effective weight management, enhances insulin sensitivity, and reduces insulin resistance which are pivotal in the therapeutic management of MetS.

### Lipid-related metabolism

LDL and VLDL are the principal lipid carriers in the bloodstream. VLDL is synthesized in the liver and plays a crucial role in transporting triglycerides to peripheral tissues. In the context of obesity, the apolipoprotein C-II (Apo C-II) present on the VLDL surface activates lipoprotein lipase, promoting the hydrolysis of triglycerides. Subsequently, a significant proportion of VLDL is converted into LDL. An excessive accumulation of LDL, particularly oxidized LDL, predisposes to induce arteriosclerosis (27). A short-term VLCD effectively modulates the metabolism of LDL and VLDL, thereby contributing to the management of MetS and reducing cardiovascular risk. Additionally, glycerol serves as a critical intermediate in fatty acid metabolism. By regulating glycerol metabolism and reducing fat accumulation, a short-term VLCD treatment offers an effective therapeutic approach for addressing MetS.

### Insulin sensitivity-related metabolism

Leucine and valine are metabolized within muscle tissue, where they not only generate energy and promote muscle mass augmentation but also stimulate insulin secretion. Nevertheless, their metabolic intermediates carry the potential to induce insulin resistance (25, 28). It has been demonstrated that regulating branched-chain amino acid (BCAA) metabolism can alleviate insulin resistance (29). Glycine enhances insulin secretion by activating receptors on pancreatic β-cells (30), while serine modulates insulin sensitivity in the liver (31). Glutamine facilitates the release of glucagon-like peptide-1 and boosts insulin secretion (32), whereas glutamate can elevate glucagon secretion and worsen insulin resistance (33, 34). A short-term VLCD treatment significantly modulates the levels of these relevant metabolites. By optimizing their metabolic profiles, this dietary intervention may enhance insulin sensitivity and mitigate insulin resistance in patients with MetS.

### Inflammation and oxidative stress-related metabolism

Glycine mitigates the levels of pro-inflammatory cytokines through the regulation of NF-κB, while methylhistidine exerts an inhibitory effect on pro-inflammatory cytokines (35, 36). Glutamine influences glycolytic processes and the subsequent inflammatory response (37), and glutamate modulates inflammatory signaling pathways through its specific receptors (38). Betaine participates in methylation reactions, thereby reducing the production of inflammatory mediators (39). Glutathione, a tripeptide composed of glutamic acid, cysteine, and glycine, exhibits potent antioxidant properties (40). Betaine enhances non-enzymatic antioxidant defenses, further contributing to cellular protection (41). MetS is intricately associated with chronic inflammation and oxidative stress (42–44). The short-term VLCD treatment significantly modulates the levels of these pertinent metabolites, indicating its potential anti-inflammatory and antioxidant effects. This observation is consistent with previous studies demonstrating that caloric restriction diets reduce inflammatory cytokine levels and mitigate oxidative damage. Collectively, these findings underscore the multiple metabolic pathways through which a short-term VLCD treatment contributes to the amelioration of MetS (45, 46).

### Neurotransmitter-related metabolism

Glutamate functions as the predominant excitatory neurotransmitter in the central nervous system, significantly influencing the release of neurohormones (47). Glutamine plays a pivotal role in the synthesis and transmission of glutamate, ensuring its availability for neurotransmission. Betaine can modulate the methylation processes of neurohormone receptors, thereby affecting their function and expression (48). MetS is closely associated with dysregulation of neurohormones, which are crucial for maintaining energy balance, regulating appetite, and orchestrating other physiological processes (49). The administration of a short-term VLCD treatment effectively modulates the levels of these relevant metabolites. By optimizing neurohormone signaling pathways, this dietary intervention exerts a beneficial effect on the management and alleviation of MetS.

In conclusion, the metabolic profiles associated with MetS underwent significant alterations, with short-term VLCD treatment effectively regulating characteristic metabolites involved in energy metabolism, lipid metabolism, insulin sensitivity, inflammation, oxidative stress, cell signaling, and neurotransmitter-related pathways. Furthermore, short-term VLCD treatment may improve anthropometric parameters, blood pressure, and lipid metabolism indices in MetS patients by modulating multiple metabolites.

In this study, the observed alterations cannot be solely ascribed to the short-term VLCD treatment for MetS, since factors such as time effects, regression to the mean, or medication withdrawal may also play a role. It should be noted that the sample size in this study was relatively limited. In the future, a 9-month follow-up observation will be established to assess the durability of therapeutic efficacy and its long-term effects on the cardiovascular system. Moreover, 50 to 100 participants will be enrolled, and targeted quantitative techniques applied to further validate the results of this study.

## Data Availability

The raw data supporting the conclusions of this article will be made available by the authors, without undue reservation.
